# Supplementary material for: Cover cropping can be a stronger determinant than host crop identity for arbuscular mycorrhizal fungal communities colonizing maize and soybean
Source: PeerJ. 2019 Feb 8;7:e6403. doi: 10.7717/peerj.6403 (PMC6369830; doi:10.7717/peerj.6403)
Supplement: Supplemental Information 1 [file peerj-07-6403-s001.pdf]

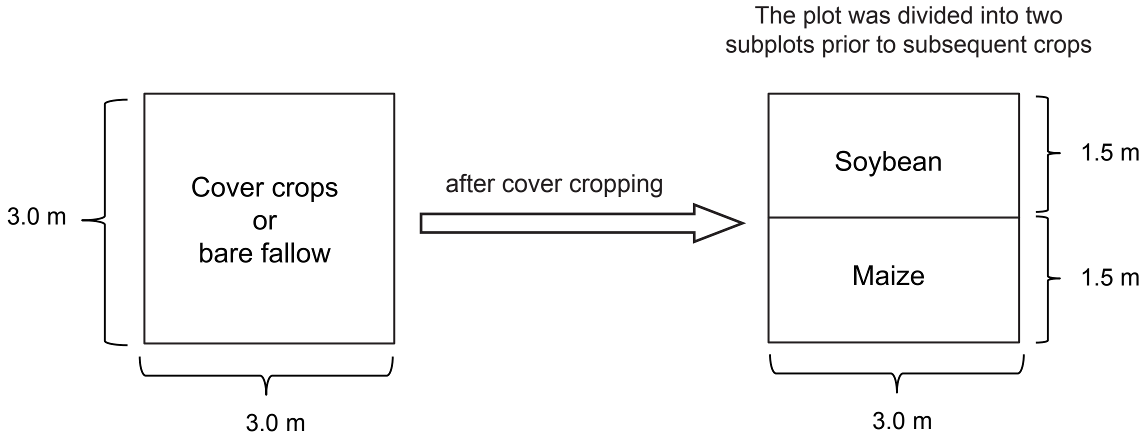

Cover crop managements	Sowing cover crops	Subsequent host crops	Sowing subsequent crops
Italian ryegrass	November 9, 2016	Soybean and maize	May 29, 2017
Hairy vetch	November 9, 2016	Soybean and maize	May 29, 2017
Brown mustard	November 9, 2016	Soybean and maize	May 29, 2017
Bare fallow	—	Soybean and maize	May 29, 2017
