# Supplementary figures and images for: Cover cropping can be a stronger determinant than host crop identity for arbuscular mycorrhizal fungal communities colonizing maize and soybean
Source: PeerJ. 2019 Feb 8;7:e6403. doi: 10.7717/peerj.6403 (PMC6369830; doi:10.7717/peerj.6403)

A

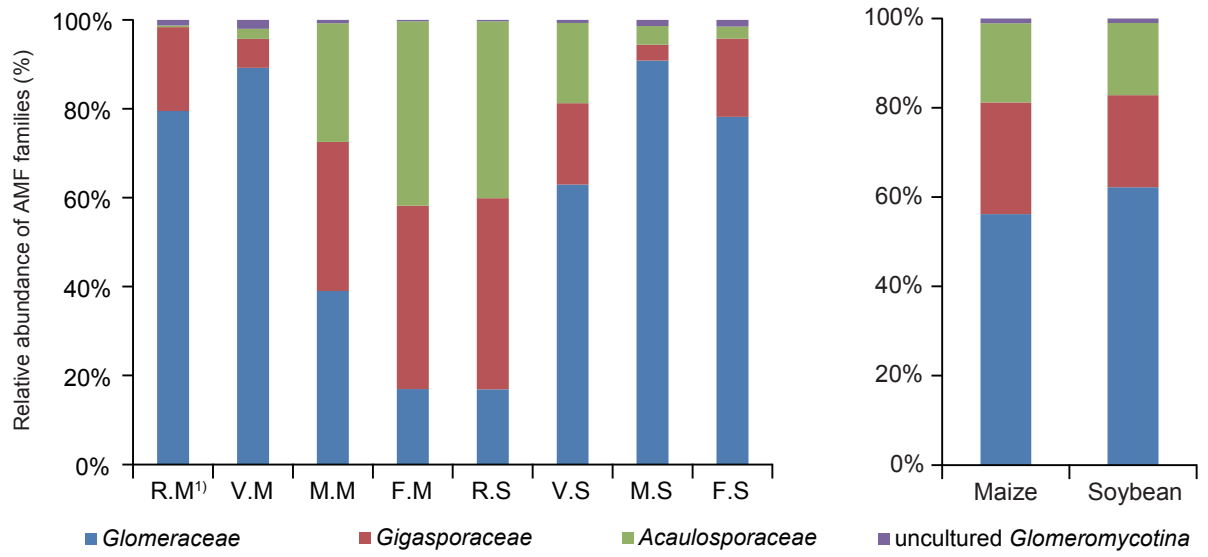

B

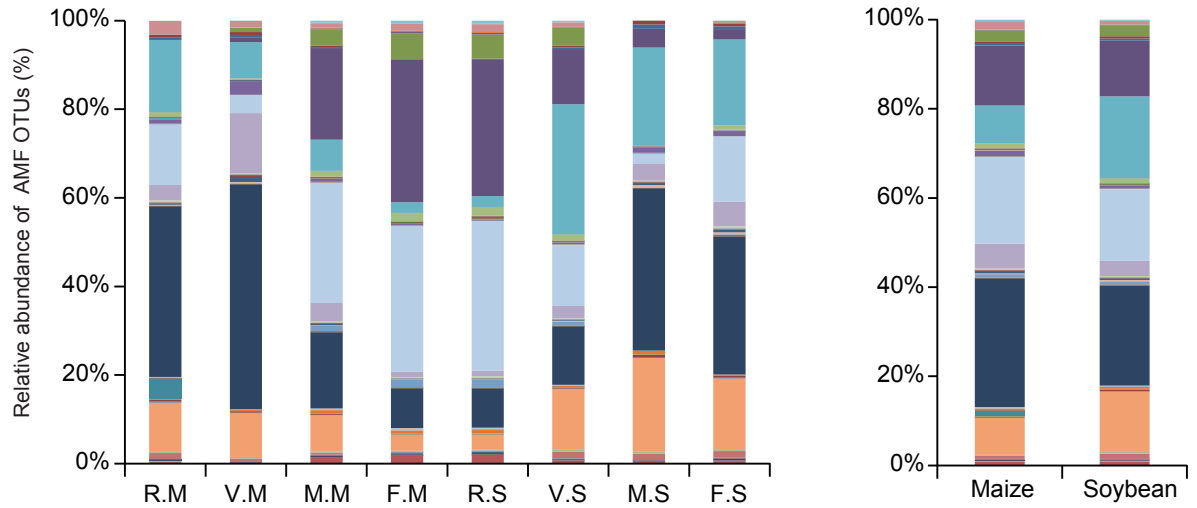

Supplement: Supplemental Information 2 — 1) R.M, V.M, M.M and F.M show the relative abundance of OTU in maize after cultivation of Italian ryegrass, hairy vetch, brown mustard cropping or bare fallow, respectively. R.S, V.S, M.S and F.S show the relative abundance of OTU in soybean after cultivation of Italian ryegrass, hairy vetch, brown mustard cropping or bare fallow, respectively. A = Family-based abundance, B = OTU-based abundance. [file peerj-07-6403-s002.pdf]
